# Enzyme-Catalyzed Synthesis of Unsaturated Aliphatic Polyesters Based on Green Monomers from Renewable Resources

**DOI:** 10.3390/biom3030461

**Published:** 2013-08-12

**Authors:** Yi Jiang, Albert J.J. Woortman, Gert O.R. Alberda van Ekenstein, Katja Loos

**Affiliations:** 1 Department of Polymer Chemistry, Zernike Institute for Advanced Materials, University of Groningen, Nijenborgh 4, 9747 AG Groningen, The Netherlands; E-Mails: Y.Jiang@rug.nl (Y.J.); A.J.J.Woortman@rug.nl (A.J.J.W.); G.O.R.Alberda.van.Ekenstein@rug.nl (G.O.R.A.E.); 2 Dutch Polymer Institute (DPI), P.O. Box 902, 5600 AX Eindhoven, The Netherlands

**Keywords:** enzymatic polymerizations, CALB, unsaturated aliphatic polyesters, bio-based monomers, succinate, itaconate, 1,4-butanediol, renewable resources

## Abstract

Bio-based commercially available succinate, itaconate and 1,4-butanediol are enzymatically co-polymerized in solution *via* a two-stage method, using *Candida antarctica* Lipase B (CALB, in immobilized form as Novozyme® 435) as the biocatalyst. The chemical structures of the obtained products, poly(butylene succinate) (PBS) and poly(butylene succinate-*co*-itaconate) (PBSI), are confirmed by ^1^H- and ^13^C-NMR. The effects of the reaction conditions on the CALB-catalyzed synthesis of PBSI are fully investigated, and the optimal polymerization conditions are obtained. With the established method, PBSI with tunable compositions and satisfying reaction yields is produced. The ^1^H-NMR results confirm that carbon-carbon double bonds are well preserved in PBSI. The differential scanning calorimetry (DSC) and thermal gravimetric analysis (TGA) results indicate that the amount of itaconate in the co-polyesters has no obvious effects on the glass-transition temperature and the thermal stability of PBS and PBSI, but has significant effects on the melting temperature.

## 1. Introduction

Utilizing renewable resources for the replacement of depleting fossil stocks is an appealing research topic, both in the academic and industrial areas [1,2,3,4,5]. It is a promising approach to solve the severe environmental problems induced by the increasing petroleum consumptions nowadays and the plausible energy shortage in the future. As abundant carbon-neutral renewable resources, biomass stocks are generated directly from solar energy in a short cycle. A great number of monomers and macromonomers can be produced from biomass stocks by natural biological activities or chemical modifications [6,7]. These bio-based monomers provide numerous opportunities for the synthesis of green and novel polymers.

Unsaturated polyesters are widely used as thermosetting resins in various industrial areas [8,9,10]. They are usually produced by polycondensation of diacids and diols based on petroleum stocks, using titanium or tin alkoxides as catalyst [11]. The synthesis temperature is usually above 150 °C [11,12]. Many monomers with chemically or thermally unstable moieties are not suitable for polyester synthesis, due to uncontrollable side reactions induced by such a high temperature, like gelation, decomposition and discoloration [11,12,13]. Besides, the residual metals from the conventional catalysts are hard to remove, which may cause undesirable pollution upon disposal [12].

*Candida antarctica* Lipase B (CALB) is a very versatile biocatalyst for polyester synthesis, working with various monomers and organic solvents under mild conditions [12,14,15,16,17,18,19,20,21,22,23,24,25,26,27]. CALB-catalyzed synthesis of polyesters from green monomers has recently gained increasing popularity. Succinate [28,29,30,31], fatty acids from plant oils [11,32,33,34,35,36,37], isosorbide [29,30] and 1,4-butanediol [8,28,31,38,39,40] are extensively studied. The bio-based polyesters produced are eco-friendly, since the monomers and the catalysts are all generated from renewable resources, and the polymers are biodegradable [9,28,39].

Itaconate is a commercially available green bio-based monomer. Its acid derivative, itaconic acid, has been industrially fermented from carbohydrates using *Aspergillus terreus* since the 1960s [41]. This monomer has interesting photoactive and biocompatible properties [9]. It is an ideal building block for constructing unsaturated polyesters with potential biomedical and engineering applications [9,42,43]. However, up until now, itaconate has not been well studied for polyester synthesis, neither by conventional catalysts nor by biocatalysts. Limited kinds of itaconate-based polyesters were synthesized using conventional chemical catalysts [8,9,10,43,44,45,46,47,48]. Only two papers referred to the enzymatic polymerizations of itaconate with other monomers: Barrett *et al*. reported enzymatic co-polymerizations of dimethyl itaconate and adipic acid with 1,4-cyclohexanedimethanol and poly(ethylene glycol) [9]; and Rajkhowa *et al*. reported Lipase-catalyzed polymerization of diglycidyl ether of bisphenol A and itaconic anhydride [49]. To the best of our knowledge, the enzyme-catalyzed co-polymerization of succinate, itaconate and 1,4-butanediol has not yet been studied. This is probably due to the low enzyme polymerizability of itaconate, which is caused by its short chain length [28] and the stereo-hindrance effect of the carbon-carbon double bond suspended around the carbonyl group. We believe CALB is the perfect biocatalyst for the synthesis of itaconate-based polyesters, due to its wide monomer adaptability, rendering polyesters in which the thermal unstable carbon-carbon double bonds could be preserved, since the enzymatic polymerization will be performed at mild temperatures under 100 °C [12,17].

**Figure 1:**
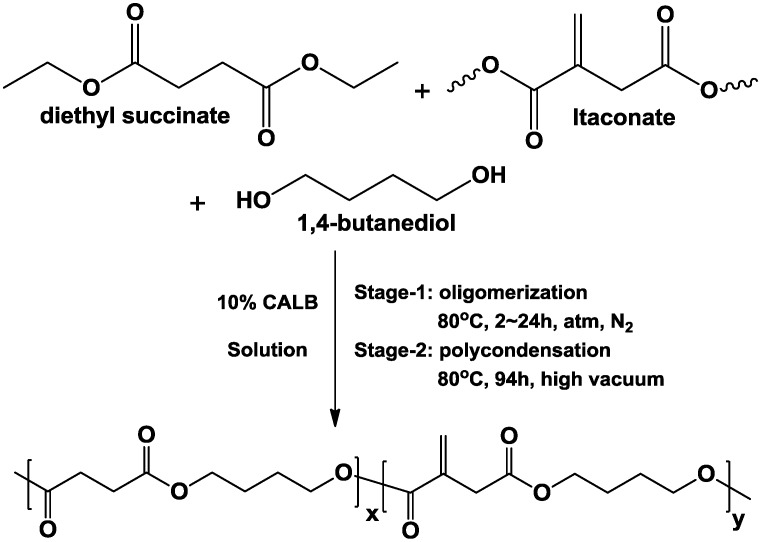
*Candida antarctica* Lipase B (CALB)-catalyzed co-polymerization of succinate, itaconate and 1,4-butanediol.

We present an environmental friendly approach towards unsaturated aliphatic polyesters. Bio-based succinate, itaconate and 1,4-butanediol are enzymatically co-polymerized in solution *via* a two-stage method, using CALB as the catalyst. The general synthesis strategy is illustrated in Figure 1. Monomers are oligomerized at 80 °C under nitrogen atmosphere during the first stage. Then, the oligomers are polycondensed at the same temperature under high vacuum during the second stage. To achieve the best polymerization results, the effects of different reaction conditions on CALB-catalyzed synthesis of poly(butylene succinate-*co*-itaconate) (PBSI) are extensively investigated, and the optimal polymerization conditions are obtained. With the method we established, poly(butylene succinate) (PBS) and a series of PBSI are synthesized. The chemical structures, molecular weight and thermal properties of the co-polyesters are characterized by different methods.

## 2. Results and Discussion

### 2.1. Effects of Polymerization Conditions on *CALB*-Catalyzed Co-Polymerization of Diethyl Succinate, Dimethyl Itaconate and 1,4-Butanediol

Succinic acid, itaconic acid and 1,4-butanediol are commercially available bio-based monomers. They are good building blocks for polyester synthesis. However, succinic acid has a rather low solubility in 1,4-butanediol under enzymatic polycondensation conditions, which leads to low polymerization efficiency [28]. Itaconic acid has the same problem, according to our observations reported in Section 2.2. To avoid phase separation during polymerization, diethyl succinate and dimethyl itaconate were used as the acyl donors.

For the purpose of producing PBSI with the highest amount of itaconate, molecular weight and reaction yield, the effects of several polymerization conditions on CALB-catalyzed co-polymerization of diethyl succinate, dimethyl itaconate and 1,4-butanediol were investigated, including solvent, solvent dosage, oligomerization time during the first stage and vacuum during the second stage.

#### 2.1.1. Effect of Solvent on CALB-Catalyzed Co-Polymerization of Diethyl Succinate, Dimethyl Itaconate and 1,4-Butanediol

Dodecane, diethylene glycol dimethyl ether (diglyme) and diphenyl ether were evaluated, as they have been proven to be suitable for enzymatic synthesis of polyesters [13,28,50]. As summarized in Table 1, PBSI with the highest mole percentage of itaconate (X_I_) and number average molecular weight (M_n_) was synthesized using diphenyl ether as solvent. However, the highest reaction yield was obtained in diglyme; the second highest reaction yield in diphenyl ether.

As presented in Table 1, when the molar feed ratio of dimethyl itaconate was 15%, the values of product X_I_ obtained in diphenyl ether, dodecane and diglyme were 10.5%, 3.5% and 0.3%, respectively. The corresponding M_n_ calculated from ^1^H-NMR spectra were 1,304 g/mol, 963 g/mol and 854 g/mol. The reaction yields were 55.5%, 54.3% and 68.8%, respectively.

Meanwhile, when the feed ratio of dimethyl itaconate was increased to 25%, the values of product X_I_ achieved in diphenyl ether, dodecane and diglyme were 15.9%, 3.7% and 5.9%, respectively. The corresponding M_n_ were 1,403 g/mol, 730 g/mol and 795 g/mol. The reaction yields were 22.6%, 18.3% and 30.2%, respectively.

**Table 1:** The effect of solvent on CALB-catalyzed synthesis of poly(butylene succinate-*co*-itaconate) (PBSI).

		Molar composition							
		Feed (%)			PBSI ^a^ (%)			Molecular weight	
Solvent	log *P*	F_S_	F_I_	F_B_	X_S_	X_I_	X_B_	M_n_ (g/mol) ^b^	Yield (%)
Diphenyl ether	4.1	35	15	50	40.5	10.5	49.0	1,304	55.5
Dodecane	6.8	35	15	50	46.5	3.5	50.0	963	54.3
Diglyme	−1.3	35	15	50	49.2	0.3	50.5	854	68.8
Diphenyl ether	4.1	25	25	50	35.3	15.9	48.8	1,403	22.6
Dodecane	6.8	25	25	50	46.5	3.7	49.8	730	18.3
Diglyme	−1.3	25	25	50	43.2	5.9	50.9	795	30.2

F_S_, F_I_, F_B_: molar feed ratio of succinate, itaconate and 1,4-butanediol; X_S_, X_I_, X_B_: mole percentage of succinate, itaconate and butylene units in PBSI; polymerization conditions: 200 wt% of solvent; Stage-1: 80 °C, 24 h, N_2_; Stage-2: 80 °C, 94 h, vacuum 40 mmHg; ^a^ molar composition determined by integration of the ^1^H-NMR spectra; ^b^ number average molecular weight (M_n_) calculated from ^1^H-NMR spectra; log *P*, logarithm of partition coefficient.

It has already been reported that diphenyl ether is the preferred solvent to achieve higher M_n_ for lipase-catalyzed synthesis of polyesters [13,28,50], which is in good accordance with our current results. Three factors could be attributed to our case, including the log *P* (logarithm of partition coefficient) value of the solvent, the accessibility of CALB and the miscibility of the intermediate and the final products in the solvent. The log *P* values of diphenyl ether and dodecane are higher than 1.9. They are more efficient for producing high molecular weight polyesters than diglyme. Furthermore, we found that CALB dispersed well in diphenyl ether and diglyme, but adhered tightly to the flasks in dodecane. Additionally, PBS with low molecular weight and PBSI are fully miscible in diphenyl ether at 80 °C, but precipitated fast in diglyme and dodecane. Therefore, PBSI with higher M_n_ is produced in diphenyl ether, since CALB is more accessible and the intermediate products are diffused better, which provide sufficient time and space for the chain growth and the transesterification of PBSI.

Moreover, we found that the enzymatic polymerizability of dimethyl itaconate was higher in diphenyl ether in comparison to the other two solvents. In addition, the molar composition of PBSI produced in diphenyl ether matched better with the feed composition of the monomers. The enzymatic polymerizability of dimethyl itaconate in dodecane and diglyme was quite low; only less than 6% of dimethyl itaconate was co-polymerized, although the reaction yield was considerably high. This is because PBS and PBSI are not soluble in dodecane and diglyme at 80 °C. In diglyme and dodecane, 1,4-butanediol prefers to react with diethyl succinate first, since the enzyme polymerizability of diethyl succinate is higher than that of dimethyl itaconate. Low molecular weight PBS and PBSI composed with a little amount of itaconate was produced and precipitated fast from the reaction.

Therefore, diphenyl ether is the most suitable solvent for CALB-catalyzed synthesis of PBSI.

#### 2.1.2. Effect of Solvent Dosage on CALB-Catalyzed Co-Polymerization of Diethyl Succinate, Dimethyl Itaconate and 1,4-Butanediol

**Figure 2:**
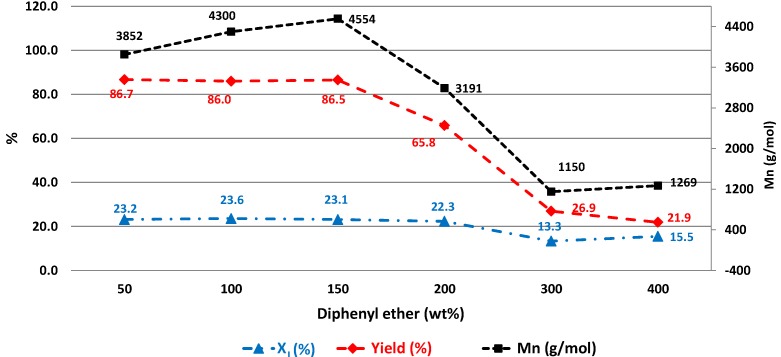
The effect of diphenyl ether dosage on CALB-catalyzed synthesis of PBSI.

Figure 2 illustrates the values of product M_n_, X_I_ and reaction yield as a function of diphenyl ether dosage. PBSI with the highest values of M_n_, X_I_ and reaction yield was obtained from the reaction with 150 wt% of diphenyl ether (in relation to the total amount of monomers).

Polymerization conditions: 50%–400 wt% of diphenyl ether; F_S_/F_I_/F_B_ = 25%/25%/50%; Stage-1: 80 °C, 2 h, N_2_; Stage-2: 80 °C, 94 h, vacuum 2 mmHg; M_n_ was calculated from ^1^H-NMR spectra.

As presented in Figure 2, the values of product X_I_ and reaction yield remained almost the same, when the dosage of diphenyl ether was increased from 50% to 150%. The product X_I_ was around 23%, and the reaction yield was close to 87%. The corresponding M_n_ increased from 3,852 g/mol to 4,554 g/mol. The X_I_ value calculated from ^1^H-NMR was in good agreement with the feed ratio of itaconate. The synchronous increase of product M_n_ with the solvent dosage was due to the better diffusion of the reactants in dilute reaction.

By increasing the solvent dosage from 150% to 400%, the values of product M_n_ and reaction yield decreased significantly. The M_n_ reduced from 4,554 g/mol to 1,269 g/mol, while the reaction yield decreased from 86.5% to 21.9%. As for the X_I_ value, it remained similar, around 23%, when the solvent dosage was increased to 200%. On further dilution to 300%, it dropped to 13.3%, and on dilution to 400%, it remained more or less the same: 14.6%. We suspect that the increase of the residual alcohol amount and the decrease of the polymerization rate in dilute reaction could be the reasons. The alcohols were more difficult to remove by vacuum at a certain low concentration in dilute solution. The absolute amount of the residual alcohols was higher in the reactions with higher solvent dosage. At the same time, the polymerization rate was reduced, since the concentrations of CALB and the reactants were lower. The transesterification of oligomers with itaconate was hindered, and only low molecular weight co-polyesters were produced. The low molecular weight PBSI composed with a higher amount of itaconate is soluble in methanol. In this case, only PBSI with a lower amount of itaconate was obtained after purification, which led to the lower value of the reaction yield.

As a result, the best diphenyl ether dosage for *in vitro* synthesis of PBSI is 150 wt% (in relation to the total amount of monomers).

#### 2.1.3. Effect of Oligomerization Time during the First Stage on CALB-Catalyzed Co-Polymerization of Diethyl Succinate, Dimethyl Itaconate and 1,4-Butanediol

As shown in Table 2, the selected oligomerization time has no obvious effects on the composition and the reaction yield of PBSI. PBSI with the highest M_n_ was synthesized from the reaction conducting 2 h oligomerization during the first stage.

**Table 2:** The effect of oligomerization time on CALB-catalyzed synthesis of PBSI.

	Molar composition							
	Feed (%)			PBSI ^a^ (%)			Molecular weight	
Oligomerization time	F_S_	F_I_	F_B_	X_S_	X_I_	X_B_	M_n_ (g/mol) ^b^	Yield (%)
2 h	35	15	50	35.7	14.1	50.2	3,936	87.7
6 h	35	15	50	35.2	14.9	49.9	3,212	87.6
12 h	35	15	50	36.3	14.4	49.3	2,960	84.1
2 h	25	25	50	27.0	23.1	49.9	2,935	73.1
6 h	25	25	50	28.9	21.5	49.6	2,459	68.5
12 h	25	25	50	27.5	22.8	49.7	2,609	71.6

Polymerization conditions: 150 wt% of diphenyl ether; Stage-1: 80 °C, 2–12 h, N_2_; Stage-2: 80 °C, 94 h, vacuum 2 mmHg; ^a^ molar composition determined by integration of the ^1^H-NMR spectra; ^b^ number average molecular weight calculated from ^1^H-NMR spectra.

When the feed ratio of dimethyl itaconate was 15%, the composition of PBSI agreed well with the feed composition of the monomers, which was independent of the time of oligomerization. The reaction yield was quite satisfying (higher than 84%). However, the product M_n_ decreased from 3,936 g/mol to 2,960 g/mol, with increasing the oligomerization time from 2 h to 12 h.

Similar trends were obtained when the feed ratio of dimethyl itaconate was 25%. In spite of increasing the oligomerization time from 2 h, 6 h and up to 12 h, the composition and reaction yield of PBSI remained almost the same. The product M_n_, however, decreased a little from 2,935 g/mol (2 h oligomerization) to 2,609 g/mol (12 h oligomerization).

**Figure 3:**
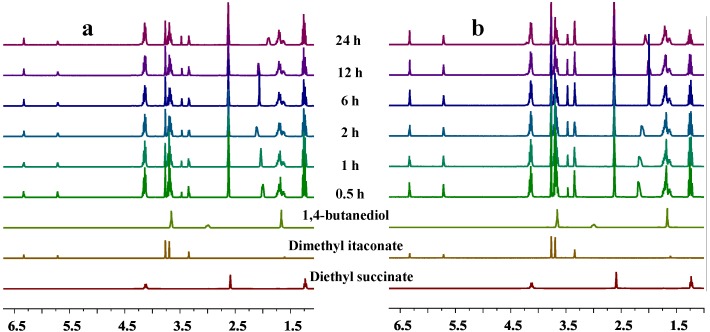
*In situ* ^1^H-NMR investigation of the oligomerization process of: (**a**) diethyl succinate (35%), dimethyl itaconate (15%) and 1,4-butanediol (50%); (**b**) diethyl succinate (25%), dimethyl itaconate (25%) and 1,4-butanediol (50%).

The enzymatic oligomerization process of diethyl succinate, dimethyl itaconate and 1,4-butanediol was monitored by *in situ* ^1^H-NMR, as shown in Figure 3. The ^1^H-NMR spectra of the intermediate products reacted for more than 30 min were almost identical, except the signals belonging to the active hydroxyl groups at 1.7–2.2 ppm. It is suspected that the monomers were fully transformed to oligomers after 30 min during the first stage.

Therefore, it can be concluded that 2 h of oligomerization during the first stage will be sufficient enough for CALB-catalyzed synthesis of PBSI.

#### 2.1.4. Effect of Vacuum during the Second Stage on CALB-Catalyzed Co-Polymerization of Diethyl Succinate, Dimethyl Itaconate and 1,4-Butanediol

As presented in Table 3, the vacuum during the second stage has a significant impact on the CALB-catalyzed co-polymerization. PBSI with the highest values of X_I_, M_n_ and reaction yield was produced by the co-polymerizations with the highest vacuum applied (2 mmHg). Furthermore, the composition of PBSI produced under higher vacuum matched better with the feed composition of the monomers.

**Table 3:** The effect of vacuum on CALB-catalyzed synthesis of PBSI.

	Molar composition							
	Feed (%)			PBSI ^a^ (%)			Molecular weight	
Vacuum	F_S_	F_I_	F_B_	X_S_	X_I_	X_B_	M_n_ (g/mol) ^b^	Yield (%)
2 mmHg	35	15	50	35.7	14.1	50.2	3,936	87.7
10–20 mmHg	35	15	50	37.1	13.1	49.8	2,552	56.2
40 mmHg	35	15	50	40.5	10.5	49.0	1,304	55.5
2 mmHg	25	25	50	27.0	23.1	49.9	2,935	73.1
10–20 mmHg	25	25	50	31.7	19.1	49.2	2,328	19.5
40 mmHg	25	25	50	35.2	15.9	48.9	1,203	22.6

Polymerization conditions: 150 wt% of diphenyl ether; Stage-1: 80 °C, 2 h, N_2_; Stage-2: 80 °C, 94 h, vacuum at 2–40 mmHg; ^a^ molar composition determined by integration of the ^1^H-NMR spectra; ^b^ number average molecular weight calculated from ^1^H-NMR spectra.

When the feed ratio of dimethyl itaconate was 15%, the values of product X_I_, M_n_ and reaction yield increased significantly with an increase of vacuum. The values of product X_I_ from the reactions under vacuum of 40 mmHg, 10–20 mmHg and 2 mmHg were 10.5%, 13.1% and 14.1%, respectively. The corresponding product M_n_ was 1,304 g/mol, 2,552 g/mol and 3,936 g/mol. The reaction yield was 55.5%, 56.2% and 87.7%, respectively.

The same trend of product X_I_, M_n_ and reaction yield as a function of vacuum was identified, when the feed ratio of dimethyl itaconate was 25%. By lowering the pressure from 40 mmHg to 2 mmHg, the values of product X_I_ increased from 15.9% to 23.1%. The product M_n_ increased from 1,203 g/mol to 2,935 g/mol. Additionally, the reaction yield increased from 22.6% to 73.1%.

The effect of vacuum is quite reasonable, since the residual alcohols and water can be further eliminated from the reaction under higher vacuum, which facilitates the chain growth of co-polyesters. It is obvious that the reduced pressure shall be regulated to 2 mmHg during the second stage for *in vitro* synthesis of PBSI in diphenyl ether.

### 2.2. Effect of Itaconate Structure on *CALB*-Catalyzed Synthesis of PBSI in Diphenyl Ether

Four itaconate derivatives were studied, namely itaconic acid, dimethyl itaconate, diethyl itaconate and dibutyl itaconate. The byproducts generated during polycondensation are water, methanol, ethanol and n-butyl alcohol, respectively. 

Phase separation of itaconic acid in the presence of the other reactants was observed. This is due to the fact that itaconic acid has a rather low solubility in the mixture of diphenyl ether, diethyl succinate and 1,4-butanediol. 

Dimethyl itaconate dissolved in the reaction medium when at temperatures above 60 °C. The co-polymerization with dimethyl itaconate, diethyl itaconate or dibutyl itaconate was homogeneous.

**Figure 4:**
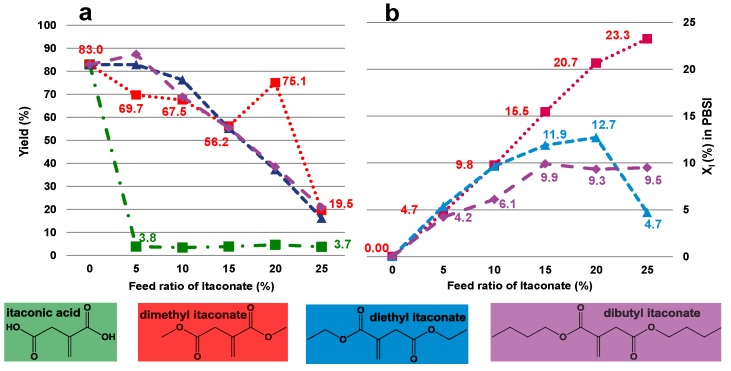
The effect of itaconate structure on CALB-catalyzed synthesis of PBSI: (**a**) the reaction yield of PBSI as a function of the feed ratio of itaconate; (**b**) the mole percentage of itaconate in PBSI (X_I_) as a function of the feed ratio of itaconate.

PBSI could not be obtained in a sufficient amount when using itaconic acid as the unsaturated monomer. The reaction yield was extremely low, less than 4%, as shown in Figure 4a. 

Polymerization conditions: 150 wt% of diphenyl ether; Stage-1: 80 °C, 2 h, N_2_; Stage-2: 80 °C, 94 h, vacuum 10–20 mmHg.

For the co-polymerizations with dialkyl itaconate esters, the reaction yield of PBSI decreased with increasing the feed ratio of itaconate, as displayed in Figure 4a, e.g., the reaction yield decreased from 83.0% to 19.5% when the feed ratio of dimethyl itaconate increased from 0% to 25%. The same trend of the reaction yield as a function of the feed ratio of itaconate was also observed in other co-polymerizations with diethyl itaconate or dibutyl itaconate.

Moreover, for reactions with dimethyl itaconate, the value of product X_I_ was in good accordance with the corresponding feed ratio of itaconate, as presented in Figure 4b. While the feed ratio of itaconate was 0%, 5%, 10%, 15%, 20% and 25%, the value of product X_I_ was 0.0%, 4.7%, 9.8%, 15.5%, 20.7% and 23.3%, respectively. The deviation between the X_I_ value calculated from ^1^H-NMR spectra and the corresponding feed ratio of itaconate was quite small. 

However, for co-polymerizations with diethyl itaconate, the value of product X_I_ deviated obviously from the corresponding feed ratio of itaconate, especially when the feed ratio of diethyl itaconate was higher than 10%. While the feed ratio of diethyl itaconate was 0%, 5%, 10%, 15%, 20% and 25%, the corresponding product X_I_ was 0.0%, 5.4%, 9.7%, 11.9%, 12.7% and 4.7%, respectively. The value of product X_I_ only matched with the feed ratio of diethyl itaconate when less than or equal to 10% of diethyl itaconate was added. The highest X_I_ value was only 12.7%, which was produced from the reaction with 20% of diethyl itaconate. 

For co-polymerizations with dibutyl itaconate, the highest X_I_ value achieved was only 9.9%. The value of product X_I_ increased from 0.0% to 9.9% as the corresponding feed ratio of dibutyl itaconate ascended from 0% to 15%. A plateau value was reached of around 10% when the feed ratio of dibutyl itaconate was further increased from 15% to 25%. 

As a result, the enzyme polymerizability sequence of itaconate derivatives in diphenyl ether is dimethyl itaconate, diethyl itaconate, dibutyl itaconate and itaconic acid, from high to low. Itaconic acid is not favored by CALB, since it is immiscible in solution, which makes it inaccessible to the biocatalyst. Dimethyl itaconate is the most polymerizable derivative by CALB, since the reaction byproduct, methanol, can be removed easily under vacuum, because of the low boiling temperature of 64.7 °C, compared with ethanol (bp = 78.4 °C) and n-butyl alcohol (bp = 117.7 °C). Besides, the chemical reactivity of the alkyl esters is another reason. It is well known that methyl esters are chemically much more reactive than ethyl and butyl esters.

Therefore, dimethyl itaconate is the most preferred unsaturated monomer for *in vitro* synthesis of PBSI in diphenyl ether.

In conclusion, combining the results from Section 2.1 to Section 2.2, we have established the optimal polymerization conditions for CALB-catalyzed co-polymerization of diethyl succinate, itaconate and 1,4-butanediol, which are as follows:

(1) using diphenyl ether as solvent;
(2) the dosage of diphenyl ether is 150 wt% (in relation to the total amount of monomers);
(3) oligomerization for 2 h during the first stage;
(4) regulating vacuum to 2 mmHg during the second stage;
(5) applying dimethyl itaconate as the unsaturated monomer.

### 2.3. *CALB*-Catalyzed Synthesis of PBSI Using Optimal Polymerization Conditions

Diethyl succinate, dimethyl itaconate and 1,4-butanediol were co-polymerized in the presence of CALB, using the optimal conditions we established. A series of co-polyesters was produced by alternating the feed ratio of dimethyl itaconate from 0% to 30%. No products were obtained if the feed ratio of dimethyl itaconate was increased to 35% and 50%. For the two control reactions without CALB, also no polymers were obtained after purification. The molar composition, reaction yield, molecular weight and the thermal properties of PBS and PBSI are summarized in Table 4.

**Figure 5:**
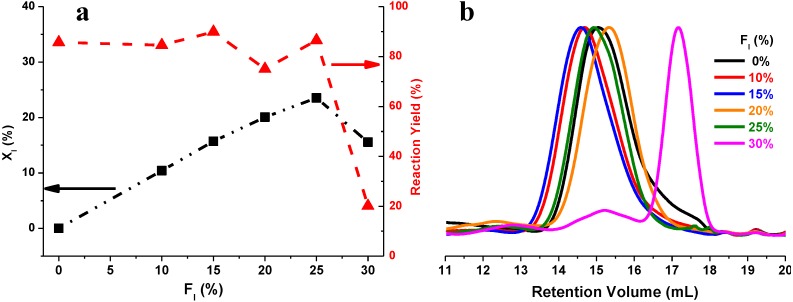
(**a**) The mole percentage of itaconate (X_I_) and the reaction yield of PBSI as a function of the feed ratio of dimethyl itaconate (F_I_); (**b**) Overlay of gel permeation chromatography (GPC) elution curves in chloroform; PBSI was synthesized *via* the optimal conditions.

As shown in Table 4 and plotted in Figure 5a, co-polyesters with tunable compositions and satisfying reaction yields were achieved, by adjusting the feed ratio of dimethyl itaconate from 0% to 25%. The mole percentage of itaconate in PBSI can be controlled from 0% to 23.5%. The corresponding reaction yield was quite good, higher than 75%.

**Table 4:** Summary of the results for the CALB-catalyzed synthesis of PBS and PBSI.

	Molar composition										Molecular Weight			
	Feed (%)			PBSI ^a^ (%)							NMR	GPC		
Co-polyester	F_S_	F_I_	F_B_	X_S_	X_I_	X_B_	Yield (%)	T_g_^b^ (°C)	T_m_^c^ (°C)	T_d_^d^ (°C)	M_n_^e^	M_n_	M_w_	M_w_/M_n_
**PBS**	50	0	50	50.5	0.0	49.5	85.7	−35.9	112.9	406.1	4,463	6,017	11,520	1.91
**PB_50_S_40_I_10_**	40	10	50	39.7	10.4	49.9	84.6	−38.8	94.6	402.0	4,696	10,128	19,236	1.90
**PB_50_S_35_I_15_**	35	15	50	35.0	15.7	49.3	90.0	−36.7	80.3	410.9	6,494	13,288	22,642	1.70
**PB_50_S_30_I_20_**	30	20	50	30.9	20.1	49.0	75.1	−37.5	71.2	404.7	5,670	8,394	11,892	1.42
**PB_50_S_25_I_25_**	25	25	50	27.2	23.5	49.3	86.5	−38.4	57.4	407.9	4,554	11,096	16,587	1.49
**PBSI**	20	30	50	36.4	15.6	48.0	20.9	−49.6	69.8	396.9	1,004	1,948	2,203	1.13
**NA ^f^**	15	35	50	-	-	-	-	-	-	-	-	-	-	-
**NA ^f^**	0	50	50	-	-	-	-	-	-	-	-	-	-	-
**Control-1 ^g^**	50	0	50	-	-	-	-	-	-	-	-	-	-	-
**Control-2 ^g^**	25	25	50	-	-	-	-	-	-	-	-	-	-	-

^a^ Molar composition determined by integration of the ^1^H-NMR spectra; ^b^ glass-transition temperature (T_g_) from differential scanning calorimetry (DSC) by a quench method; ^c^ melting temperature (T_m_) from DSC measurements, first scan; ^d^ the temperature of the maximal rate of decomposition (T_d_) from thermal gravimetric analysis (TGA) measurement; ^e^ number average molecular weight calculated from ^1^H-NMR spectra; ^f^ no polymer was obtained after precipitation; ^g^ control reactions without CALB. No polymer was obtained after precipitation.

**Figure 6:**
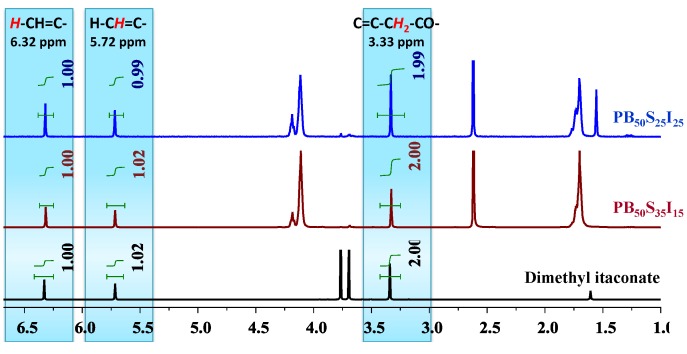
^1^H-NMR spectra of dimethyl itaconate, PB_50_S_35_I_15_ and PB_50_S_25_I_25_ in CDCl_3_.

The carbon-carbon double bonds were well preserved in the unsaturated co-polyesters, as confirmed by ^1^H-NMR spectra in Figure 6. No resonances can be assigned to the deterioration of carbon-carbon double bonds. As displayed in the ^1^H-NMR spectra of PB_50_S_35_I_15_ and PB_50_S_25_I_25_, the ratio between the integration values of the two separated peaks assigned to the protons of the carbon-carbon double bonds (6.32 ppm and 5.72 ppm, respectively) and the peak assigned to the protons of the methylene group of itaconate (3.33 ppm) was close to 1:1:2, which was exactly the same as that of dimethyl itaconate. Besides, the final products were white semicrystalline powders without discoloration. It is clear that no side reaction occurred during the enzymatic co-polymerizations.

The product M_n_ calculated from ^1^H-NMR was around 4,000–6,500 g/mol, while the feed ratio of dimethyl itaconate was less than or equal to 25%. GPC results indicate that the corresponding M_n_ was around 6,000–14,000 g/mol. The polydispersity index (PDI, M_w_/M_n_) was between 1.42 and 1.91. The molecular weight of the saturated polyesters was lower than that of the unsaturated counterparts, and the PDI was higher. This is due to the higher melting temperature of PBS, which is around 112 °C. During the polycondensation step, the reaction with PBS solidified after 19 h at 80 °C. The chain growth and transesterification of PBS stopped in the solid state. In contrast to this, the reaction with PBSI proceeded continuously in the liquid state.

The highest value of X_I_ achieved in PBSI was 23.5%, from the reaction with 25% of dimethyl itaconate. If the feed ratio of dimethyl itaconate was increased to 30%, the product X_I_ reduced to 15.6%. The corresponding product M_n_ and the reaction yield decreased significantly to 1,948 g/mol and 20.9%, as shown in Figure 5a. If the feed ratio of dimethyl itaconate was further increased to 35% and 50%, no polymer was obtained after precipitation in cold methanol.

As shown in Figure 5b, PBSI synthesized from the reaction with 30% of dimethyl itaconate had two peaks. The first peak was at a retention volume between 13.6 to 16.2 mL, similar to that of the co-polyesters with high molecular weight; however, the concentration was much lower. The major peak was at a retention volume between 16.2 to 18.2 mL, which means that the molecular weight of the most PBSI produced was quite low. It is clear that co-polyesters with two different molecular weight distributions were produced from reaction with 30% of dimethyl itaconate.

**Figure 7:**
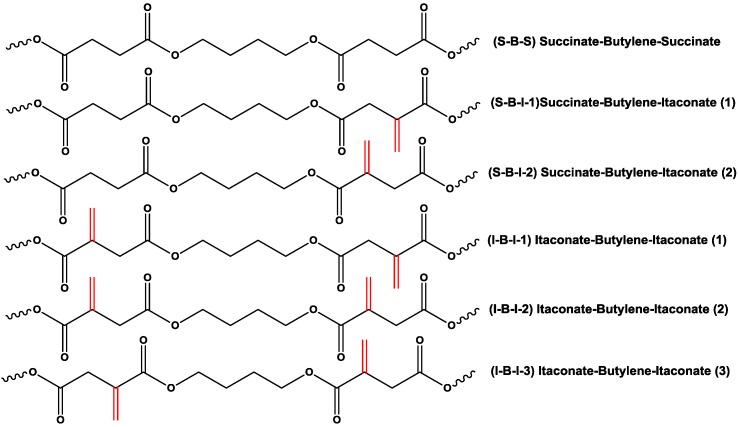
Possible types of microstructures in PBSI, the formation sequence from easy to difficult: S-B-S < S-B-I-1 < S-B-I-2 < I-B-I-1 < I-B-I-2 < I-B-I-3.

The limited incorporation of itaconate in PBSI could be attributed to the relatively low enzyme polymerizability of dimethyl itaconate compared to diethyl succinate. As shown in Figure 7, there are six possible microstructures in PBSI. It is easier for CALB to produce the microstructure of succinate-butylene-succinate (S-B-S) than that of succinate-butylene-itaconate (S-B-I) and itaconate-butylene-itaconate (I-B-I). The formation sequence from easy to difficult is S-B-S, S-B-I-1, S-B-I-2, I-B-I-1, I-B-I-2 and I-B-I-3. While the feed ratio of itaconate is 25%, the molar ratio between succinate, itaconate and 1,4-butanediol is 1:1:2. The possible dominant microstructures in PBSI could be -(S-B-I-B)_n_-. If the feed ratio of itaconate is higher than 25%, the possible structures could be -I-B-(S-B-I-B)_n_-I-B-, while some extra I-B-I structures are formed. However, the formation of the I-B-I microstructure is quite difficulty. Oligomers with itaconate ends will be terminated in chain growth, and the transesterification of oligomers with I-B-I structures will be hindered. Consequently, the molecular weight of PBSI will be lower. Low molecular weight PBSI is soluble in methanol and will be washed away during the purification steps. This is why we obtain only PBSI with the highest mole percentage of itaconate around 25%.

This hypothesis also explains well why PBSI obtained from the reaction with 30% of dimethyl itaconate had lower values for M_n_, mole percentage of itaconate and reaction yield, as well as, had two retention volume peaks, as plotted in Figure 5.

### 2.4. Thermal Properties of PBS and PBSI

**Figure 8:**
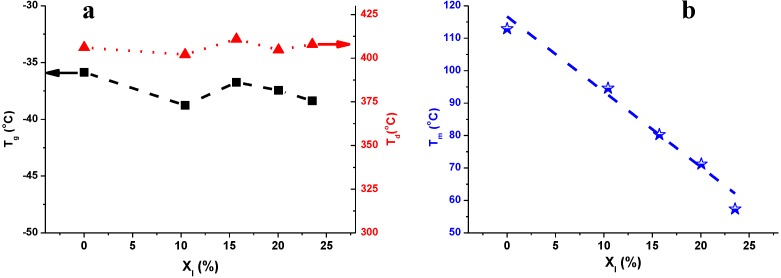
(**a**) the T_g_ and T_d_ of PBSI as a function of the mole percentage of itaconate (X_I_); (**b**) the T_m_ of PBSI as a function of the mole percentage of itaconate (X_I_); PBSI was synthesized *via* the optimal conditions.

The glass transition temperature (T_g_), the melting temperature (T_m_) and the temperature of the maximal rate of decomposition (T_d_) of PBS and PBSI are plotted as a function of the mole percentage of itaconate in Figure 8. We found that the amount of itaconate composed in the co-polyesters has no obvious effects on the T_g_ and the thermal stability of PBSI. The T_g_ and T_d_ of PBS and PBSI were similar, as shown in Figure 8a. The T_g_ of the saturated PBS was −35.9 °C. It was slightly higher than that of the rest of the unsaturated PBSI, which was around −36–−38 °C. The T_d_ of all polyesters was around 400 °C. 

However, the T_m_ of the co-polyesters is significantly affected by the amount of itaconate composed in PBSI. The T_m_ of the saturated PBS was 112.9 °C, which was 55.5 °C higher than that of the unsaturated counterpart with 23.5% of itaconate. The T_m_ of PBSI decreased almost linearly as a function of the mole percentage of itaconate, as shown in Figure 8b.

## 3. Experimental

### 3.1. Materials

Lipase acrylic resin from *Candida antarctica* Lipase B (CALB, in immobilized form as Novozyme® 435, 5,000+ U/g), diethyl succinate (99%), itaconic acid (99%+), dimethyl itaconate (99%), 1,4-butanediol (99%+), dodecane (99%+, anhydrous), diethylene glycol dimethyl ether (99.5%, anhydrous) and diphenyl ether (99%) were purchased from Aldrich. Diethyl itaconate (98%+) and dibutyl itaconate (95%+) were purchased from TCI EUROPE. Diphenyl ether was vacuum distilled and stored with 4Å molecular sieves before use. The other chemicals were used as received.

### 3.2. General Procedure for *CALB*-Catalyzed Co-Polymerization of Succinate, Itaconate and 1,4-Butanediol in Solution

CALB (10 wt% in relation to the total amount of monomers, for all reactions) was first fed into a 25 mL flask and stored in a desiccator with phosphorus pentoxide at room temperature under high vacuum for 16 h. Then diethyl succinate (5.55 mmol), itaconate (5.55 mmol), 1,4-butanediol (11.10 mmol) and the solvent (usually 150 wt% in relation to the total amount of monomers) were added. With the protection of nitrogen, the monomers were oligomerized at 80 °C for 2–24 h under atmospheric pressure during the first stage. The oligomers were further polymerized for another 94 h at the same temperature under the reduced pressure of 2–40 mmHg during the second stage.

After polymerization, chloroform (25 mL) was added into the reaction flask. CALB was filtered out and washed with chloroform (35 mL) three times. The collected solution was condensed by rotary evaporation at 40 °C under a vacuum of 300 mbar. The products were precipitated in cold methanol (−20 °C) and washed with methanol three times. Finally, the polyesters were dried in a vacuum oven at 40 °C for 2–3 days before analysis. The final polymers obtained were white semicrystalline powders.

**Figure 9:**
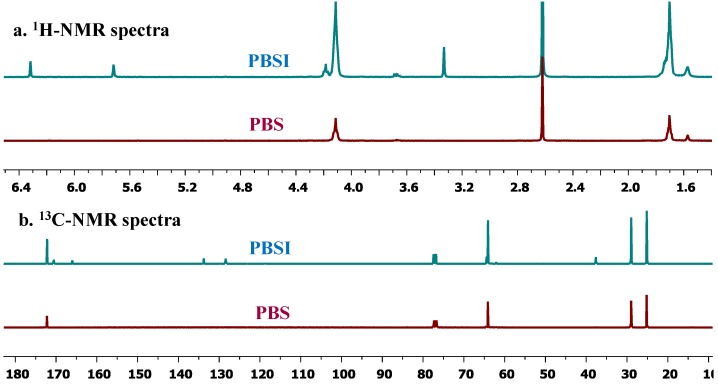
(**a**) ^1^H-; (**b**) ^13^C-NMR spectra of PBS and PBSI.

^1^H-NMR (400 MHz, CDCl_3_, ppm) analysis of PBS and PBSI: 6.25–6.35 (1H, s, ***H***-CH=C-CO-, from itaconate), 5.65–5.75 (1H, s, H-C***H***=C-CO-, from itaconate), 4.05–4.25 (4H, m, -CO-O-C***H***_2_-, from 1,4-butanediol), 3.30–3.40 (2H, s, -C=C-C***H***_2_-CO-, from itaconate), 2.58–2.68 (4H, s, -OC-C***H***_2_-, from succinate), 1.60–1.78 (4H, m, -O-CH_2_-C***H***_2_-C***H***_2_-CH_2_-O-, from 1,4-butanediol); low intensity signals due to end group-groups were observed at 3.75–3.79 (3H, s, -O-C***H***_3_, from itaconate), 3.63–3.73 (2H, m, -C***H***_2_-OH, from 1,4-butanediol) and 1.15–1.32 (3H, m, -OCH_2_C***H***_3_, from succinate). The spectra are shown in Figure 9a.

^13^C-NMR (100 MHz, CDCl_3_, ppm) analysis of PBS and PBSI: 172.2 (-***C***O-, from succinate), 170.6 (-***C***O-, from itaconate), 166.0 (-C=C-***C***O-O-, from itaconate), 133.8 (-***C***=C-, from itaconate), 128.4 (-C=***C***-, from itaconate), 62.5–65.0 (-CO-***C***H_2_-, from 1,4-butanediol), 37.6 (-***C***H_2_-, from itaconate), 29.0 (-***C***H_2_-, from succinate), 25.2 (-***C***H_2_-, from 1,4-butanediol); low intensity signals assigned to end-groups were determined at 62.2 (-***C***H_2_-, from 1,4-butanediol) and 51.8 (-***C***H_3_, from itaconate). The spectra are shown in Figure 9b.

### 3.3. Control Reactions for Enzymatic Polymerization

Diethyl succinate, dimethyl itaconate and 1,4-butanediol were co-polymerized without CALB using the same method as we established. No polymer was obtained after precipitation.

### 3.4. *In situ* ^1^H-NMR Investigation of the Oligomerization Process of Diethyl Succinate, Dimethyl Itaconate and 1,4-Butanediol in Diphenyl Ether

CALB (10 wt% in relation to the total amount of monomers) was pre-dried according to the general procedure described in Section 3.2. Diphenyl ether (150 wt% in relation to the total amount of monomers) was used in this study. Diethyl succinate, dimethyl itaconate and 1,4-butanediol were enzymatically oligomerized at 80 °C under atmospheric pressure with the protection of nitrogen for 24 h. At pre-selected time intervals, about 20 mg of the solution was withdrawn from the reaction. They were added directly into a NMR tube containing 1 g of CDCl_3_ for ^1^H-NMR analysis.

### 3.5. Instrumental Methods

^1^H- and ^13^C-NMR spectra of PBS and PBSI were characterized on a Varian VXR spectrometer (400 MHz for ^1^H-NMR analysis and 100 MHz for ^13^C-NMR analysis), using CDCl_3_ as solvent. The chemical shifts reported were referenced to the resonances of tetramethylsilane (TMS) or the solvent. 

The molecular weight (M_n_ and M_w_) and the polydispersity index (PDI) were measured by gel permeation chromatography (GPC) using a Viscotek GPC equipped with three detectors (LS detector: Viscotek Ralls detector; VS detector: Viscotek Viscometer Model H502; RI detector: Shodex RI-71 Refractive Index detector), using a guard column (PLgel 5 µm Guard, 50 mm) and two columns (PLgel 5 µm MIXED-C, 300 mm, from Agilent Technologies) at 30 °C. Chloroform of HPLC grade was used as the eluent at a flow rate of 1.0 mL/min. The molecular weight calculations were performed based on the universal calibration. Narrow polydispersity polystyrene standards (Agilent and Polymer Laboratories), with a weight-average molecular weight from 645 to 3,001,000 g/mol, were used to generate the universal calibration curves. 

The glass transition temperature and the melting temperature of the co-polyesters were measured by differential scanning calorimetry (DSC) using a TA-Instruments Q1000 DSC. A quench method was conducted for the T_g_ measurement. The heating rate was 20 °C/min.

Thermal gravimetric analysis (TGA) was performed on a Perkin Elmer Thermo Gravimetric Analyzer TGA7. Samples were measured at a scan rate of 10 °C/min under nitrogen environment.

### 3.6. Calculation of the Molar Composition of PBSI from ^1^H-NMR Spectra

**Figure 10:**
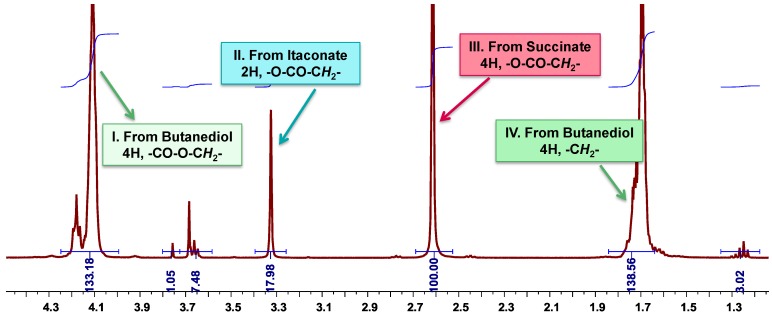
Calculation of the molar composition of PBSI from ^1^H-NMR spectra.


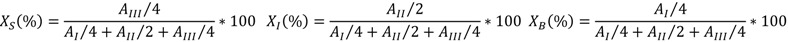
(1)

The molar composition of PBSI was calculated from ^1^H-NMR spectra, as presented in Figure 10 and Equation (1). A_I_, A_II_ and A_III_ are the integration values of Peak I, Peak II and Peak III, respectively, as displayed in Figure 10. X_S_, X_I_ and X_B_ represent the mole percentage of succinate, itaconate and butylene unit in PBSI. 

### 3.7. Calculation of the Number Average Molecular Weight of PBSI from ^1^H-NMR Spectra

**Figure 11:**
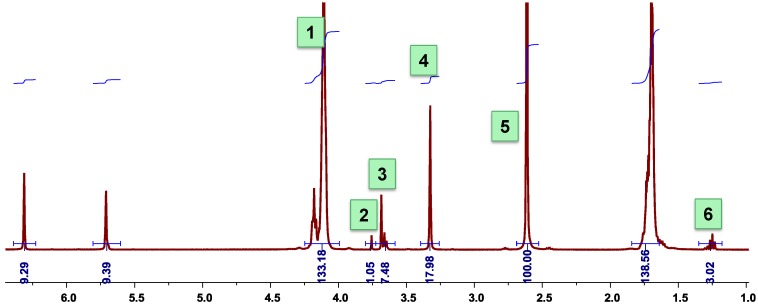
Calculation of the M_n_ from ^1^H-NMR spectra.


The number average molecular weight (M_n_) was characterized from ^1^H-NMR spectra, as shown in Figure 11 and Equation (2). I_1_, I_4_, and I_5 _ are the integration values of the peaks assigned to the backbones of PBSI, which are originated from 1,4-butanediol, itaconate and succinate, respectively, as shown in Figure 11. I_2_, I_3_ and I_6_ are the integration values of the peaks assigned to the end groups.

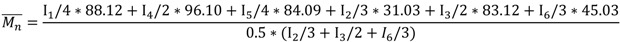
(2)

## 4. Conclusions

Fully bio-based poly(butylene succinate) and poly(butylene succinate-*co*-itaconate) were synthesized by CALB-catalyzed enzymatic co-polymerizations of succinate, itaconate and 1,4-butanediol *via* the two-stage method. It is a totally green approach toward unsaturated aliphatic polyesters, since all the building blocks and catalysts are generated from renewable resources. 

The effect of solvent on enzymatic co-polymerization was studied. We found that diphenyl ether was the most suitable solvent, resulting in the highest product M_n_ and mole percentage of itaconate, as well as satisfying reaction yields. We believe that such an effect could be attributed to the log *P* value of the solvent, the accessibility of CALB in the reaction medium and the solubility of the intermediate and final products in the solvent.

The study on the effect of diphenyl ether dosage indicated that the preferred solvent amount was 150 wt% (in relation to the total amount of monomers). The M_n_, mole percentage of itaconate and reaction yield of PBSI increased with the increase of diphenyl ether dosage first, and then, decreased. The diffusion of the reactants, the residual byproducts amount in the reaction and the polymerization rate are the three factors contributing to such an effect.

The selected oligomerization time during the first stage had no obvious effects on CALB-catalyzed synthesis of PBSI. The *in situ* NMR investigations indicated that the oligomerization process was completed after 30 min.

We found that the vacuum during the second stage had a significant influence on enzymatic polycondensation. The best results were achieved under the highest vacuum, since the equilibrium of polycondensation was further shifted to the final products by removing the residual water and alcohols.

The enzymatic polymerizability sequence of itaconate derivatives in diphenyl ether was identified, which was dimethyl itaconate, diethyl itaconate, dibutyl itaconate and itaconic acid, from high to low. The boiling temperature of the reaction byproducts and the accessibility of itaconate to the biocatalyst are two major reasons attributed to this sequence. 

With the method here, PBS and a series of PBSI were successfully synthesized with good reaction yields. The amount of itaconate in PBSI was tunable, by adjusting the feed ratio of dimethyl itaconate. The carbon-carbon double bonds of itaconate were well preserved in PBSI. The molecular weight of PBS was lower than that of the unsaturated counterparts, but the PDI was a bit higher.

The values of the product M_n_, mole percentage of itaconate and reaction yield decreased significantly with the increasing of the feed ratio of dimethyl itaconate to 30%. If it was further increased to 35%, or even higher, no product was obtained.

We found that the amount of itaconate in PBSI has no obvious effects on the glass-transition temperature and the thermal stability of the co-polyesters, but has significant effects on the melting temperature. The glass transition temperature of PBS and PBSI was similar, around −35–−38 °C. The temperature of the maximal rate of decomposition remained similar, around 400 °C. However, the melting temperature of the obtained co-polyesters decreased almost linearly as the mole percentage of itaconate in PBSI increased.
